# Cancer immunotherapies: A hope for the uncurable?

**DOI:** 10.3389/fmmed.2023.1140977

**Published:** 2023-02-17

**Authors:** Firas Hamdan, Vincenzo Cerullo

**Affiliations:** ^1^ Laboratory of Immunovirotherapy, Drug Research Program, Faculty of Pharmacy, University of Helsinki, Helsinki, Finland; ^2^ TRIMM, Translational Immunology Research Program, University of Helsinki, Helsinki, Finland; ^3^ Drug Delivery, Drug Research Program, Division of Pharmaceutical Biosciences, Faculty of Pharmacy, University of Helsinki, Helsinki, Finland; ^4^ iCAN Digital Precision Cancer Medicine Flagship, University of Helsinki, Helsinki, Finland; ^5^ Department of Molecular Medicine and Medical Biotechnology and CEINGE, Naples University Federico II, Naples, Italy

**Keywords:** cancer, immunotherapy, oncolytic virotherapy, antibodies, syngergistic effects

## Abstract

The use of cancer immunotherapies is not novel but has been used over the decades in the clinic. Only recently have we found the true potential of stimulating an anti-tumor response after the breakthrough of checkpoint inhibitors. Cancer immunotherapies have become the first line treatment for many malignancies at various stages. Nevertheless, the clinical results in terms of overall survival and progression free survival were not as anticipated. Majority of cancer patients do not respond to immunotherapies and the reasons differ. Hence, further improvements for cancer immunotherapies are crucially needed. In the review, we will discuss various forms of cancer immunotherapies that are being tested or already in the clinic. Moreover, we also highlight future directions to improve such therapies.

## 1 Introduction

The pronunciation of the word cancer may alarm the majority, if not all, of the people. This is simply because of its serious health effects which has brought it to be the second leading cause of death, after cardiac conditions (Heron, 2019; Siegel et al., 2020). Scientific surveys from the United Kingdom in 2019 have observed that the public’s main support for scientific research concerned cancer research (for Business and Strategy, 2020). Analysts perceive this wide support towards the subject as an eager awaited miracle drug that could cure cancer. Yet, as our understanding of cancer grows the discovery of such miracle drug seems to be lost hope.

Cancers have shown to have a dense complexity regarding its biology. In 2000, Hanahan and Weinberg published one of the most cited reviews in the cancer biology field describing the hallmarks of cancer (Hanahan and Weinberg, 2000). During those times six hallmarks were described to oversimplify the needed characteristics for a successful tumor growth. Based on such hallmarks, traditional therapies were developed ranging from radiation, chemotherapy, and targeted therapies.

To this day, surgery, radiation and chemotherapy represent one of the most used therapies for the treatment of cancer (Harrington and Smith, 2008). The main mode of action of these therapies is the direct tumor cell lysis *via* different mechanisms. This may be perceived as old fashioned since this mode of action has seen not to be that effective in eradicating full tumors. Moreover, these therapies have a growing list of serious side effects detrimental to patients. In spite of this and as our understanding of cancer biology grew, targeted therapies took the stage since they were more effective and safer (Baudino, 2015). Targeted therapies consist of molecules that exploit certain biological difference among healthy and cancer cells allowing for selectively targeting. Nevertheless, advancements in genomic sequencing have demonstrated that cancer is not a monogenic disease yet a complex and heterogenous disease (Meldrum et al., 2011). This explains why the use of targeted therapies targeting single molecules have not shown an overwhelming success as once expected.

After a decade from the Hanahan and Weinberg review, the authors updated the list of hallmarks by adding two new hallmarks: reprogramming energy metabolism and evading immune responses (Hanahan and Weinberg, 2011a). The latter hallmark consists of a crucial interplay where the immune system has the capabilities to recognize and kill cancer cells. Hence, tumor cells have developed multiple strategies to overcome immune recognition. Also, tumor cells have shown to induce a tumor-promoting inflammation. As a result of this, a novel era of treatments was developed enhancing the immune system to recognize cancer. In 2013, the world-renowned science journal, *Science*, dubbed such treatment as “breakthrough of the year” due to the significant impact in the clinic and in 2018 were the theme for the Nobel prize in physiology and medicine (Couzin-Frankel, 2013). These treatments offer the use of the patient’s own immune system to induce anti-tumor response able to sustain a long-lasting anti-tumor killing.

As successful as immunotherapies have been described, looking at the statistical numbers a very small minority (20%–40%) of people do benefit from them (Sharma et al., 2017). Multiple reasons have been formulated over the years concerning why most of the patients do not respond. Nevertheless, a need to improve such treatments is required. This review will provide the current state of immunotherapies and provide novel strategies to further such field.

## 2 Cancer immunotherapies

Our immune system has a significant role in keeping the integrity of our health. Besides its obvious role in protecting against pathogens, it has a more unobtrusive but highly crucial role in cancer prevention and defence. Already in 1909, Paul Ehrlich postulated that the power of the immune system may be harnessed to control cancer. It was proposed that immune cells are constantly surveilling cells throughout the body, able to recognise and eliminate incipient cancer cells and therefore halt the production of nascent tumours (Hanahan and Weinberg, 2011b). This was validated by striking results where immunocompromised individuals had an increased risk in developing certain cancers (Vajdic and van Leeuwen, 2009). Furthermore, mice models with defective T cells and NK cells were shown to be more susceptible to cancer (Smyth et al., 2000). However, according to such logic, tumours that appear and progress in otherwise healthy individuals should be able to somehow resist or evade elimination by the immune system. Further research indeed indicated that the tumour microenvironment was immunosuppressive and cancer cells are able to develop multiple immune evasion strategies (Vinay et al., 2015). In spite of this, boosting the immune system has been the major target for drug development in the treatment of cancers for the past decade.

Our immune system has a well-known ability to distinguish between self and non-self, especially in the case of infection or malignancy. This process is called immune surveillance and it is crucial in eliminating hundreds of newly formed malignant cells daily. Nevertheless, other than serving as a tumor suppressor the immune system can also shape the tumor immunogenicity in a process called cancer immunoediting. This process is divided into three parts; elimination, equilibrium and escape (Dunn et al., 2004). The first phase consists of a dynamic process in which immune cells recognize tumor cells expressing immunogenic antigens (Kim et al., 2007). This then allows the immune cells to recognize and kill tumor cells. However, not all of tumor cells are immunogenic leading them to not be recognized by immune cells. This adds a bottle neck pressure inducing a positive selection of tumor cells with reduced immunogenicity. These cells then enter the final stage of escape since they are unharmed by the immune system and can proliferate uncontrollably (Dunn et al., 2006; Kim et al., 2007; Swann and Smyth, 2007).

In spite of this, the main objective for cancer immunotherapies is to redirect the immune system towards these cells with reduced immunogenicity (Seliger, 2005). The current cancer immunotherapies in the clinic can be divided into two groups based on mechanism of actions: passive or active immunotherapies. Active immunotherapies involve the direct activation of a tumor-specific immune response. While passive immunotherapies are molecules that are given to patients that cannot either be induced, lowly expressed or non-functioning.

### 2.1 Passive immunotherapies

In many patients, the ability to induce a proper anti-tumor immune response is hindered by factors of immunosuppression. Thus, passive immunotherapies try to overcome such limitation by fighting cancer directly. These molecules endow intrinsic antitumoral activity and can indirectly or directly target tumor cells. In this section these types of molecules will be further explained.

#### 2.1.1 Cytokines

Cytokines are small molecules expressed by both inflammatory and non-inflammatory cells to coordinate inflammation and other immune responses. In cancer, these molecules have been administered to patients in order to stimulate anti-cancer immune responses in an un-specific way. Two main cytokines that will be discussed are interleukin-2 (IL-2) and granulocyte and macrophage colony stimulating factor (GM-CSF).

IL-2 is a pluripotent cytokine able to stimulate the immune system in many ways. However, one of its crucial roles is in the activation of both natural killer (NK) cells20 and T-cells (Gillis and Smith, 1977; Gillis et al., 1978; Smith, 1988; Lehmann et al., 2001). In specific, high levels of Il-2 can induce T cell expansion and activation for interferon gamma (IFN-gamma) production (Paliard et al., 1988). A recombinant form of Il-2, marketed as Proleukin^®^, has received FDA approval for the use in metastatic renal cell carcinoma (RCC) (Fyfe et al., 1995) and metastatic melanoma (Amaria et al., 2015). Clinical data has shown that from 270 metastatic melanoma patients, 16% of patients showed objective responses while 6% showed complete response (Davar et al., 2017). Similar results also were seen in metastatic RCC, objective responses were seen in 15% of patients and 8% of patients showed complete responses (Achkar et al., 2017). Moreover, a clear increase in NK and T cell activation was observed in most of the treated patients. Hence, currently in the clinic Proleukin^®^ is still being tested with other potential synergistic molecules to further improve clinical responses. Nevertheless, systemic administration of IL-2 has been associated with several life-threatening toxicities due to an increased inflammation (Pachella et al., 2015). In spite of this, several strategies are being developed to ensure a targeted release in the tumor microenvironment.

One other widely used cytokine in the clinic is GM-CSF. Compared to IL-2, GM-CSF works with other types of cells in specific APCs. For example, mice defective in GM-CSF had a decrease proliferation and maturation of dendritic cells (DC) and macrophages leading to an increase vulnerability to bacterial infections (Stanley et al., 1994). In 2005, Kurbacher *et al.* treated 19 cancer patients suffering from breast and female reproductive tract carcinomas with recombinant GM-CSF (Kurbacher et al., 2005). Only one patient had a complete response while six others had a partial response. The main mode of action was shown to be attributed to the activation of DCs and increased antigen presentation. A recombinant protein of GM-CSF (called sagramostim) showed a 100% overall response rate with chronic lymphocytic leukemia patients when combined with chemotherapy. Yet, with chronic myeloid leukemia it was discontinued in all patients due to severe adverse events.

#### 2.1.2 Adoptive cell therapy (ACT)

One other form of passive cancer immunotherapy may come in the form of infusing activated immune cells into patients. This is called adoptive cell therapy (Rosenberg et al., 2008; Perica et al., 2015) and can be divided into two subtypes: adoptive tumor-infiltrating lymphocytes (TILs) and genetically engineered T cells expressing specific T cell receptors (TCRs) or chimeric antigen receptors (CARs). Both these therapies share a common step which is the preconditioning lymphodepletion regimen before treatment. With the use of cyclophosphamide, patients undergo lymphopenia and neutropenia in order to prevent such endogenous cells from attacking injected activated immune cells (Proietti et al., 1998). Moreover, these therapies use the patient’s own lymphocytes.

TIL therapy is not a novel form of treatment but can be dated back to 1994 being used in metastatic melanoma (Rosenberg et al., 1994). This therapy consists of isolating tumor-specific T cells within the tumor microenvironment and further expand them *ex-vivo*. Various regimens for expansion have been described, but the most common is the use of high doses of IL-2. Currently such therapy has been approved by the FDA for metastatic melanoma. Yet, multiple clinical trials are undergoing with different type of expansion regimens or in combination with other treatments.

Other than TILs, scientists have tried to increase the armamentarium of T-cells by genetically engineering them to express specific TCRs or CARs (Rosenberg and Restifo, 2015). In both cases, T cells are first isolated through leukapheresis using peripheral blood. Once T cells have been isolated, using a lentiviral vector a transduction is performed to facilitate expression of a TCR or CAR. Following transductions, these cells are then expanded using high doses of IL-2 and are then ready to be re-infused in patients.

Other than the structure, TCRs and CARs give T cells a different way of killing tumor cells. TCRs are made of αβ heterodimers with each chain consisting of variable and constant region domains. These receptors associate with CD3 in the surface membrane and recognize major histocompatibility complexes (MHC) loaded with an antigen which induces activation and killing of the tumor cell (Harris and Kranz, 2016; June et al., 2018). As for CARs, these receptors consist of an antigen binding domain, consisting of single-chain variable fragment (scFv) from an antibody, which is connected to an intracellular signaling domain causing cell activation (June et al., 2018). Thus, killing of a tumor cell occurs in a MHC-independent fashion where once the scFv portion of the receptor binds to its specific epitope it triggers T cell activation *via* its signaling domain (June et al., 2018). This is a clear advantage over the conventional TCR killing since one of the most prominent immune-escape mechanisms a tumor poses the downregulation of MHC from its surface (Blankenstein et al., 2012). Yet, TCRs can recognize intracellular tumor antigens presented by MHC molecules while CAR receptors can only recognized membrane-bound tumor antigens. Moreover, CAR-based T cells are more toxic than TCR based (Restifo et al., 2012). Both forms of treatments can cause neurotoxicity and cytokine release syndrome (CRS), yet with CAR-based T cell therapy it is more severe (Restifo et al., 2012). Also, Currently, six different CAR-T cell therapies have been approved in the clinic. These CAR-T cells consists of a CAR receptors targeting CD19 or BCMA for B-cell based malignancies or multiple myeloma, respectively. As for solid-tumors, CAR-T cells have not managed to obtain clinical approval due to immunosuppressive tumor microenvironment, poor infiltration, poor tumor penetration and other reasons.

#### 2.1.3 Antibody therapy

Antibody therapy in cancer has been one of the most successful types of therapies used in the clinic to treat hematological and solid tumors. The advancement of antibodies in cancer therapy can date back to 1890 when first described as neutralizing substances against diphtheria (Behring, 2013). It was later seen that these substances had a specific property in recognizing specific epitopes and were secreted by our own cells, in specific plasma B cells (Fagraeus, 1947; Van Epps, 2005). It was then hypothesized that each plasma B cell clone was able to produce one specific antibody (Nossal and Lederberg, 1958). This concept led to the isolation of individual plasma B cell clones in order to obtain monoclonal antibodies (Schwaber and Cohen, 1973; Köhler and Milstein, 1975). This technology allowed for the screening of thousands of monoclonal antibodies to identify high-affinity monoclonal antibodies against any desired tumor-associated antigen. The first clinical trial using an antibody began in 1980 for lymphoma patients (Koprowski et al., 1978; Nadler et al., 1980). Sadly, such antibodies provided poor clinical efficacy since such antibodies were murine and induced a human anti-mouse antibody (HAMA) response. Therefore, to optimize antibody therapy techniques to humanize antibodies originating from mice were developed. These techniques included cloning the murine derived variable chains (chimeric) or complementary determining regions (humanized) into human antibody formats. Recent techniques have now allowed for the generation of full human antibodies by using transgenic mice or yeast-phage display (Riechmann et al., 1988; Nelson et al., 2010). As a result of such advancements, antibody therapy has become the most type of drug sold for pharmaceutical purposes.

Antibodies are able to directly kill target cells by disrupting or activating receptor signaling. This activity is pertinent to the Fab regions of an antibody which are responsible for binding. However other than direct cell killing, antibodies are also able to orchestrate host-immune response to induced immune-mediated cell death. This dual mechanism of action has made antibody therapy powerful and safe compared to other conventional therapies. This section will describe the structure of an antibody and its use in cancer therapy.

##### 2.1.3.1 Antibody structure

**FIGURE 1:**
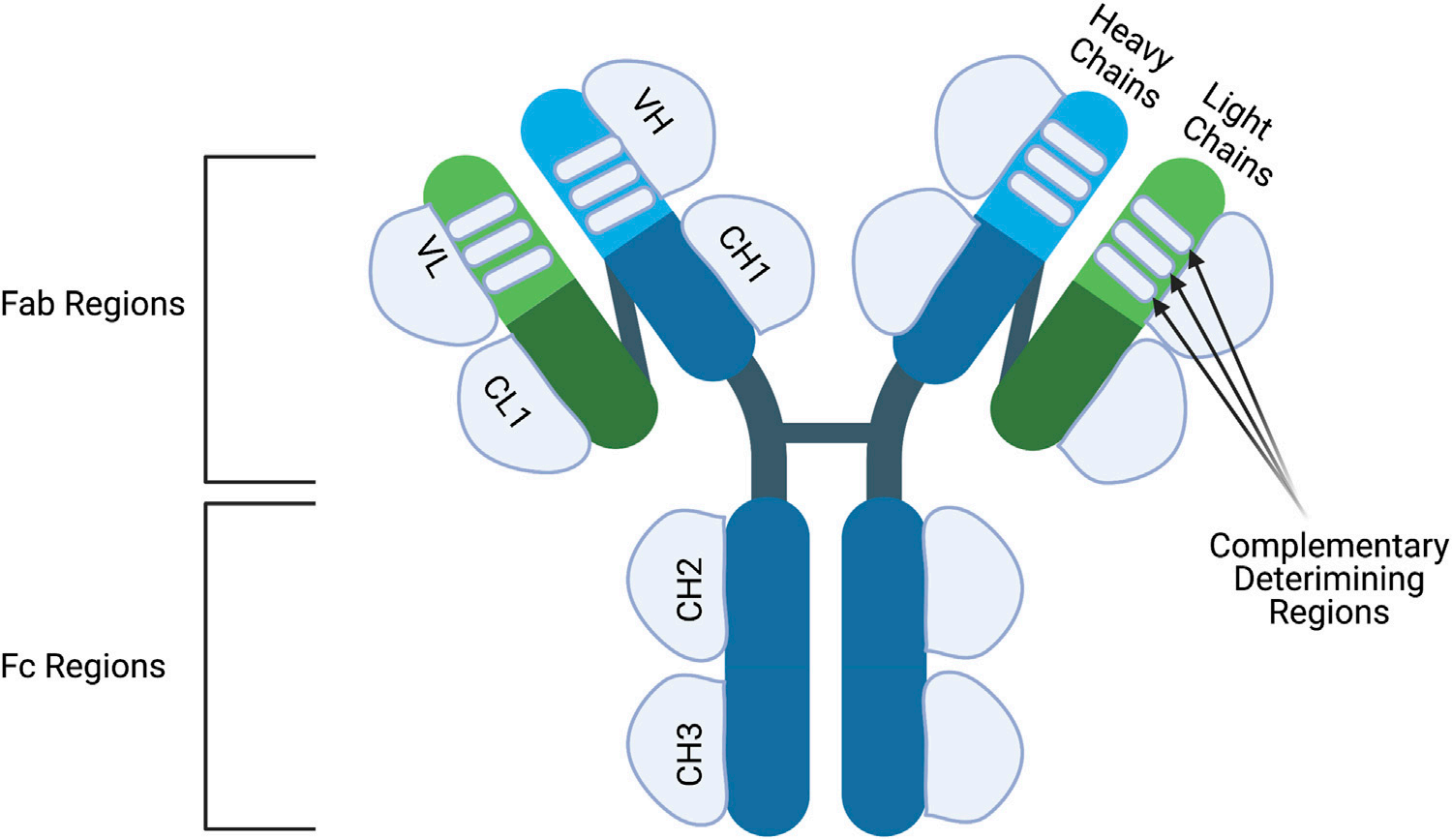
The structure of an antibody. Antibodies are made up of two identical heavy chains andlight chains. The heavy chains are connected to each other via disulfide bridge and the light chainsare connected to the upper part of the heavy chains. Both heavy and light chains consist of variable (V) and constant regions (C). The heavy chain contains three constant domains (CH1-CH3) and onevariable domain (VH) while the light chain has one constant domain (CL) and one variable domain (VL). Moreover, the variable chains have three complementary determining regions which dictatethe specificity of the antibody. Antibodies can also be classified into two structures; the Fc and Fabregion. The Fc regions contains the CH2 and CH3 domains and is important to elicit Fc-effectormechanisms. The Fab regions compromise of CH1, VH, CL1 and VL regions and are important forepitope binding. Figures were created with BioRender.com.

Antibodies are large structures made up of four polypeptides, two heavy and two light chains, joined together *via* disulfide bonds to give a “Y” shaped structure (Figure 1). Both heavy and light chains are made up of two regions: the variable and constant domains. Light chains consist of one variable domain (V_L_) and one constant domain (C_L_) while heavy chains compromise of one variable domain (V_H_) and four constant domains (C_H_1, C_H_2, C_H_3 and C_H_4). Furthermore, based on structure, antibodies can be classified into two Fab (Fragment antigen-binding) regions and an Fc (Fragment crystallizable) region. The Fab region compromises of the full light chain and part of the heavy chain (V_H_ and C_H_1) which give the tips of the “Y” shape. The rest of the constant heavy chain domains make up the Fc region which forms the stalk of the “Y” shape.

The variable regions of both the heavy and light chain are then subdivided into four framework regions and three hypervariable regions. The amino-acid composition of the hypervariable regions is the most varied from antibody-to-antibody. Once these regions fold into three β-strands they are then referred to as complementary-determining regions (CDR) since the shape complements the targeted epitope. The CDRs from the heavy and light chains determine the antibody-binding side but the framework regions also play a minor role. As for the Fc region, it compromises of C_H_2- C_H_4 and has a vital role for modulating immune cell activity. Immune effector cells can bind to the Fc-region of an antibody through the Fc-receptors subsequently activating effector functions.

##### 2.1.3.2 Heavy and light chains of antibodies

In mammals, two types of light chains of an antibody exist called lambda and kappa. No functional differences have been described for both these chains which are used to build an antibody complex. However, the antibody complex contains two identical light chains, and no mix of kappa and lambda chains usually occurs within one antibody. The proportions of each chain used varies among species and can serve as markers of abnormal proliferation of B cell clones (Hershberg et al., 2015).

**FIGURE 2:**
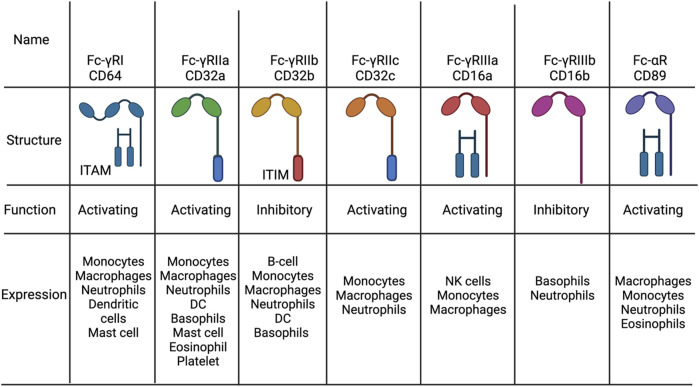
The distrubion, structure and function of Fc receptors. The different types of Fc-g and Fc-a receptors found in humans. All activating Fc receptors contain an immunoreceptor tyrosine-basedactivation motif (ITAM) while inhibitory Fc receptors have an immunoreceptor tyrosine-basedinhibitory motif (ITAM) or none. Figures were created with BioRender.com.

As for heavy chains, in mammals there exists five different chains called alpha, gamma, delta, epsilon and micro which give rise to five different antibody classes such as IgA, IgG, IgD, IgE and IgM, respectively. Contrary to light chains, the antibody classes differ in many functional activities, biological properties, and location. This is mostly due to the differential binding of different Fc-receptors since IgA, IgG or IgE bind to Fc-α, Fc-γ, or Fc-ε receptor respectively. These receptors are distinguished based on what type of immune cells express them and signaling properties, explaining the antibody class functions (Figure 2). For example, Fc-ε receptors are found on eosinophils, mast cells and basophils explaining the role of such receptors in allergic responses.

##### 2.1.3.3 Direct tumor killing using monoclonal antibodies

As previously mentioned, cancer antibodies have a dual mechanism of action consisting of either direct killing or inducing immune-mediated cell death of opsonized cancer cells. Cancer cells heavily depend on pro-tumor growth and survival signaling provided by different growth factor receptors. Antibodies can perturb such signaling by manipulating the activation or blocking ligand binding subsequently leading to cell death. An example of ligand blocking is the clinically approved Cetuximab, a monoclonal antibody binding to epidermal growth factor receptor (EGFR). EGFR is highly overexpressed on many different types of cancers and when activated can induce proliferation, migration, and invasion of tumor cells (Downward et al., 1984; Gusterson et al., 1984; Ullrich et al., 1984; Cowley et al., 1986). Cetuximab binding to EGFR has been seen to disrupt ligand binding and consequently lead to apoptosis of tumor cells (Li et al., 2005; Patel et al., 2009). Human epidermal growth factor receptor 2 (HER2) is another growth receptor overexpressed by tumor cells to sustain proliferation (Slamon et al., 1989). In specific, HER2 overexpression has been highly seen in ovarian and breast cancer (Slamon et al., 1989). Unlike EGFR, HER2 has no known ligand and is activated by heterodimerization to other growth receptors (Chen et al., 2003). Trastuzumab, a clinically approved monoclonal antibody against HER2, has been shown to disrupt this heterodimerization, consequently leading to tumor cell death (Plosker and Keam, 2006). Trastuzumab has been a clinical success in in treating HER2+ breast cancer patients (Plosker and Keam, 2006).

##### 2.1.3.4 Complement-dependent cytotoxicity

Antibodies can interact with the complement system through the Fc-region to activate the classical component cascade (Figure 2) (Melis et al., 2015; Taylor and Lindorfer, 2016). Once antibodies bind to the target ligand, the available Fc-regions are then able to bind complement protein, C1q. Hexamerization of near-by antibodies allows for efficient C1q binding, which then activates C1r and C1s (Diebolder et al., 2014; Wang et al., 2016). Activation of C1r and C1s leads to the proteolytic cleavage of C4 and C2 to initiate the complement cascade and subsequently complement dependent cytotoxicity (CDC) (Diebolder et al., 2014; Wang et al., 2016). However, only IgG and IgM antibodies are able to elicit CDC since they are the only antibody isotypes that have a C1q binding site. Nevertheless, IgA antibodies have also been observed to elicit CDC *via* the classical pathway, despite not having a C1q site (Evers et al., 2018; Evers et al., 2020). Yet, this has only been seen in B-cell lymphoma cells and the mechanism has been attributed to other receptors in the B-cell able to bind to C1q.

As an effector function, CDC has been shown to be required for *in vivo* efficacy. Mice having the genes encoding for C1q knocked out showed no clinical efficacy with anti-CD20 antibody, rituximab (Di Gaetano et al., 2003). Also, follicular lymphoma patients with known polymorphisms in the C1qA gene reducing CDC activity have been correlated with low clinical response to rituximab (Coiffier et al., 2008a). Despite these results, Fc-engineering to increase CDC activity has been extensively done and a successful example of this has been anti-CD20 antibody, ofatumumab. Ofatumumab has been engineered to have an increased ability to hexametrize and bind to C1q subsequently leading to higher CDC activity (Zhang, 2009). This enhancement translated into better clinical outcomes since ofatumumab outperformed rituximab in chronic lymphocytic leukemia patients (Coiffier et al., 2008b).

##### 2.1.3.5 Antibody-dependent cell phagocytosis

Macrophages express a variety of Fc receptors and in concrete Fc-γRII (CD32) and Fc-γRI (CD64) allowing for interaction with IgG antibodies against cancer (Figure 2) (Nimmerjahn et al., 2015). This interaction can then lead to cell death through a process called antibody-dependent cell mediated phagocytosis (ADCP) (Hubert et al., 2011). The role of ADCP in clinical efficacy has not been very well studied but there has been some evidence demonstrating a role in antibody efficacy. For example, rats having their macrophages depleted lost significant response towards monoclonal antibody therapy against colon carcinoma (Van Der Bij et al., 2010). Similar results were also shown with SCID-BEIGE mice transplanted with xenografts and treated with monoclonal antibody therapy (Overdijk et al., 2015). These specific mice do not have B or T cells and defective NK cells which then makes macrophages a primary effector immune cell and ADCP the main effector mechanism. These mice showed an *in vivo* clearance of leukemic cells when treated with daratumumab, an anti-CD38 antibody. ADCP efficacy in the clinic was also shown when 11 out of 12 of multiple myeloma patients showed ADCP when cells were cultured and treated with daratumumab *in vitro* (Overdijk et al., 2015).

A reason for why ADCP has not been so clearly correlated with antibody efficacy could be due to the expression of SIRPα and CD47 on macrophages and tumor cells, respectively. The interaction among both receptors leads to a “don’t eat me” signal which downregulates ADCP activity (Murata et al., 2018). Blockage of this axis has been shown to increase antibody therapy by enhancing ADCC activity. Currently, SIRPα and CD47 blockers are being tested in the clinic together with various antibody therapies (Murata et al., 2018).

##### 2.1.3.6 Antibody dependent cell mediated cytotoxicity

In 1965, antibody opsonized cancer cells were shown to be killed *via* a non-phagocytic mechanism, termed antibody-dependent cell mediated cytotoxicity (ADCC) (Möller, 1965). This effector mechanism can be elicited from different types of immune cells such as NK cells, neutrophils, monocytes, and eosinophils (Nimmerjahn and Ravetch, 2008a). However, the way cell death is elicited differs among cells and can range from release of cytotoxic granules, reactive oxygen species release or Fas/FasL signaling (Eischen and Leibson, 1997; Nimmerjahn and Ravetch, 2008b; De Saint Basile et al., 2010). The clinical relevance of ADCC was first described in 2000 where Clynes and colleagues showed that rituximab and trastuzumab relied on ADCC for efficacy (Clynes et al., 2000). Moreover, it was later seen that mice lacking FcγRs or certain mutations limiting ADCC did not respond to monoclonal antibody therapy (De Haij et al., 2010). Within the population, polymorphisms in Fc-γRIIA (CD32a) (Wu et al., 1997) and Fc-γRIIIA (CD16a) (Bibeau et al., 2009) have been found and described to increase IgG affinity and ADCC activity. In several clinical trials with rituximab, it was seen that patients with such polymorphisms had a better clinical response (Cartron et al., 2002; Weng and Levy, 2003; Hatjiharissi et al., 2007). Similar results were also shown with cetuximab (Rodríguez et al., 2012) and trastuzumab (Musolino et al., 2008; Boero et al., 2015) treating colorectal cancer and metastatic breast cancer, respectively. Further confirming such results, patients with higher response to trastuzumab also demonstrated higher ADCC activity compared to patients not responding (Arnould et al., 2006).

**FIGURE 3:**
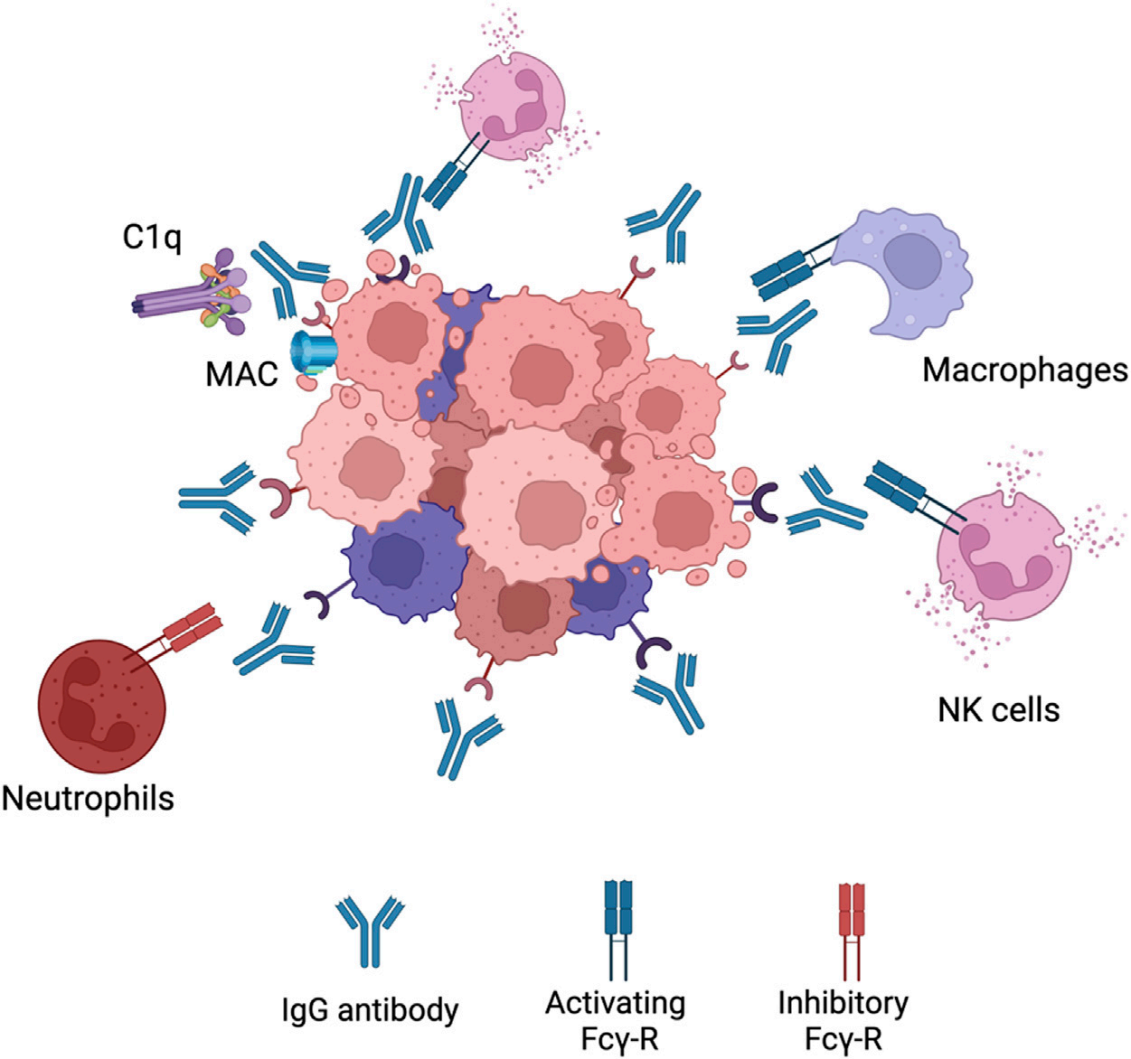
IgG1 effector mechanisms. When an IgG antibody opsonizes a cancer cell it can elicitvarious effector functions. It can interact with C1q complement protein leading to the formation ofmembrane attack complex (MAC) leading to CDC. The Fc region can also bind to activating Fcg-Rs onNK cells or macrophages to elicit ADCC or ADCP, respectively. Neutrophils express a high level ofinhibitory Fcg-Rs leading to very little activation. Figures were created with BioRender.com.

Since all the cancer antibodies in the clinic are of the IgG isotype, Fc-γRs are the main receptors that mediate ADCC. In humans there exists six different types of Fc-γRs which can be divided into activating (Fc-γRI, Fc-γRIIA, Fc-γRIIC and Fc-γRIIIA) and inhibitory (Fc-γRIIB and Fc-γRIIIB) receptors (Wallace et al., 1994). As the name indicates, the activating receptors elicit ADCC while the inhibitory receptors downregulate effector mechanisms. With IgG therapy, NK cells are the main population that elicit ADCC which is due to the type of Fc-γR expression (Figure 3). NK cells express only one Fc-γR which is the activating Fc-γRIIIA explaining its importance for ADCC mediated by IgG (Wang et al., 2015). Other myeloid and granulocytic cells also express activating Fc-γR but also higher levels of inhibitory Fc-γR. For example, inhibitory Fc-γRIIB expression on neutrophils is seven to five times higher than Fc-γRIIA (Selvaraj et al., 1988). This has been shown to have a negative role on mediating ADCC because of the competition with Fc-γRIIA (van der Kolk et al., 2002; Peipp et al., 2008). This heavy reliance on NK cell for ADCC has been shown to limit efficacy. This is because NK cells have been seen to undergo exhaustion fast and not able to elicit ADCC(97). Only 24 h later NK cells gain the ability to elicit ADCC again (Gill et al., 2012).

##### 2.1.3.7 IgA for cancer therapy

**FIGURE 4:**
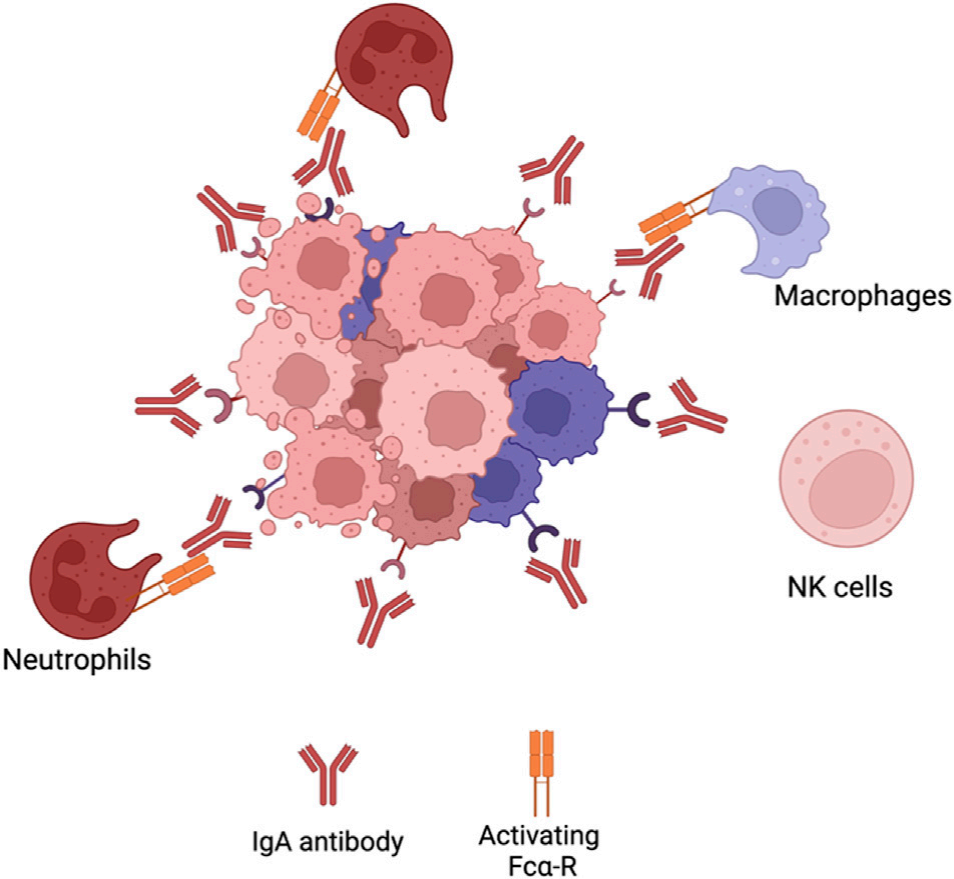
IgA effector mechanisms. IgA effector mechanisms differ from IgG antibodies. IgAantibodies do not activate CDC, since they do not have a C1q binding site. The Fc region of IgA bindsto Fca-R on neutrophils cells or macrophages to elicit ADCC or ADCP, respectively. However, since NKcells do not express Fca-R, IgA antibodies do not activate such immune population. Figures were created with BioRender.com.

Due to the limitations the IgG isotype poses, preclinical studies have been conducted on the development of cancer therapeutic mAbs with isotypes different from IgG. A potential candidate isotype is IgA (Figure 4). This antibody is the most prominent immunoglobulin isotype found in mucosal sites and the second most frequent antibody isotype in serum, after IgG (98). It consists of two different isotypes; IgA1 and IgA2 with the latter comprising three allotypes; IgA2m (Siegel et al., 2020), IgA2m (Heron, 2019) and IgA2m(n). IgA interacts with immune cells *via* binding to Fc-αR (CD89) (Pakkanen et al., 2010). Such receptor is expressed on cells of the myeloid lineage such as neutrophils, monocytes, distinct macrophage populations and eosinophils (Pakkanen et al., 2010). Initial ADCC experiments with bispecific IgG1-antibodies where one of the F (ab’)_2_ fragments was directed at the FcαR receptor and the other to a target antigen highlighted the potential use of IgA antibodies in the context of malignancies (Valerius et al., 1997). Various reports have also shown that IgA mAbs directed at different tumour antigens showed an increased ability to recruit PMNs as effector cells compared to IgG (Huls et al., 1999; Dechant et al., 2002; Lohse et al., 2011; Boross et al., 2013; Leusen, 2015). This emphasises that IgA antibodies are able to employ a distinct effector population of immune cells against tumour cells compared to IgG. Furthermore, IgA antibodies mediate macrophage dependent tumour cell killing comparable to IgG (Lohse et al., 2012). It has been suggested that IgA mAbs are not able to activate the complement system due to the lack of a C1q-binding site (Bakema and van EgmondImmunoglobulin, 2011). However, certain studies have shown that IgA antibodies (Pascal et al., 2012) or IgG-fab fragments directed against CD20107 have been able to elicit CDC of malignant B-cells through the classical pathway. The mechanism behind it is thought to be due to rearrangements in the IgM or IgG B-cell receptor (BCR) of malignant B-cells (Engelberts et al., 2016) exposing its C1q binding site mediated by the clustering of CD20 after IgA binding. The FcαR represents an advantage over FcγRs since it does not have any inhibitory receptors and no polymorphisms have been reported. This implies that more predictable responses are

Achievable with IgA. Also, the FcαR has not been implicated in shaving leading to CD20 loss. Finally, antibody internalisation occurs less frequently with IgA compared to IgG. These advantages highlight the potential use of the IgA isotype in the development of therapeutic mAbs.

##### 2.1.3.8 IgA and IgG combinational therapy for cancer

Despite the advantage of IgA, this isotype is not able to capitalize on NK cells or complement activation. To maximize on every effector population possible, scientists have tested whether combining IgG and IgA enhanced tumor killing. Bradsma and colleagues showed that using both IgG1 and IgA1 antibodies directed at different TAAs (Tumor Associated Antigens) induced higher killing than the individual antibodies when NK cells and neutrophils were present (Brandsma et al., 2015). However, when the IgG and IgA antibodies were directed towards the same TAA this enhanced effect was not seen. It is hypothesized that it could be due to the competition towards the same TAA leading to one isotype dominating in binding. Further building on this work, TrisomAB was then developed which consisted of an IgG1 antibody directed towards a TAA and Fc-αR (Heemskerk et al., 2021). TrisomAB was shown to increase tumor killing when both NK cells and neutrophils were present. This data then further supports the use of both antibody isotypes in the treatment of cancer. Similar to TrisomAB, an Fc-fusion peptide against PD-L1 with a chimeric Fc presenting both IgG1 and IgA1 heavy chains was previously described (Hamdan et al., 2021). This Fc-fusion peptide was also shown to activate effector mechanisms of both an IgG1 and IgA1 which resulted in higher tumor killing *in vitro, in vivo* and in patient-derived organoids.

##### 2.1.3.9 Fc-fusion peptides

Antibodies are large complexes which make them very hard to diffuse into large tumors (Strohl and Knight, 2009). Moreover, production is very complex and costly which inflate the price in the clinic (Strohl and Knight, 2009). Regarding such issues, a novel type of antibody-based therapeutics has been developed compromising of peptides fused to an Fc region (Nelson and Reichert, 2009; Strohl, 2009; Beck and Reichert, 2011). These peptides can be of very small size and bind to any desired target. However, when these peptides are administered systemically, they have a very short half-life due to rapid renal filtration. Attaching Fc-regions, in specific of the IgG region, increases the half-life of the peptides due to binding of Fc-neonatal receptors (Suzuki et al., 2010). Moreover, the Fc-regions can also provide Fc-effector mechanisms such as CDC, ADCC or ADCP. Currently, there are 13 different types of Fc-fusion peptides approved in the clinic used for thrombocytopenia, kidney transplants and inflammatory diseases such as arthritis or psoriasis (Strohl, 2009). Currently, no Fc-fusion peptides have been approved for the treatment of cancer. However, many Fc-fusion peptides against cancer have been described and entered clinical testing. For example, a bispecific peptide fused to a Fc of an IgG against HER-1 or HER-2 has showed high anti-tumor efficacy (Sioud et al., 2015). Moreover, the IgG Fc portion was able to elicit Fc-effector mechanisms of a normal IgG antibody.

### 2.2 Active immunotherapy

In contrast to passive, active immunotherapies are molecules that are used to induce or revitalize anti-tumor responses *in vivo.* This then requires patients to have an active and responsive immune system for successful treatment.

#### 2.2.1 Checkpoint inhibitory therapy

In the thymus, the life of a T cell begins by proliferating and creating a diverse repertoire of TCRs. In order to maintain homeostasis, the immune system needs to distinguish between self and non-self. T-cells go through an initial selection process called central tolerance. In this process T-cells that strongly react to self-peptides, presented by thymocytes, undergo apoptosis. T-cells that weakly respond to self-peptides are released as naive cells to circulate into secondary lymphoid organs. APCs, specifically dendritic cells (DC), are then able to present naive T-cells either with foreign antigens (under infection conditions), overexpressed antigens, neoantigens or mutated self-proteins (under malignancy conditions) resulting in T-cell activation. However, some of the activated T-cells have TCRs which are still able to cross-react with self-antigens. To prevent cross-reactivity to self, multiple checkpoint pathways are present during the steps of activation to prevent autoimmunity. Also, checkpoints prevent the immune system to over activate during the course of infection. This process has been termed peripheral tolerance and two main constituents that take the centre of this process are the membrane receptors CTLA-4 and PD-1. Although CTLA-4 and PD-1 have a common function, they are present in different stages of T-cell activation. CTLA-4 is called the “leader” of the immune checkpoints since it regulates the activation of naive T-cells in lymph nodes. Contrary to CTLA-4, PD-1 acts in later stages since it regulates already activated T-cells in the peripheral tissues.

##### 2.2.1.1 The CTLA-4 axis

**FIGURE 5:**
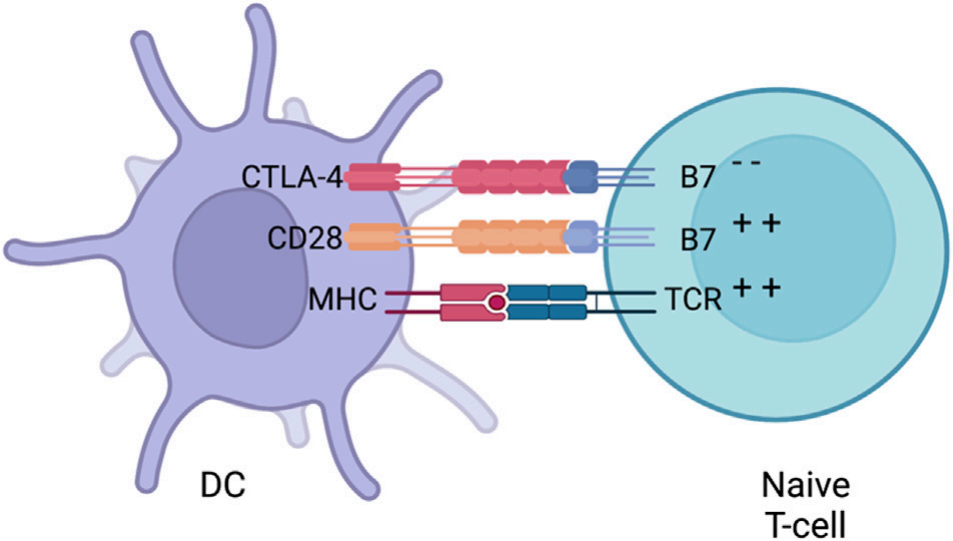
CTLA-4 suppression. Naive T-cells migrate to lymph nodes to become activated. Activationis usually provided by both MHC (loaded with an antigen) and co-stimulation from B7 (interacting with CD28) provided by a DC. After early stimulation, CTLA-4 is translocated to the surface of DCswhich then competes with B7 to bind to CD28 and downregulates T-cell activation. Whether a naiveT-cells undergoes activation or anergy is dependent on the balance of between CD28:B7 andCD28:CTLA4 signalling. Figures were created with BioRender.com.

Activating naive T-cells in the thymus is a complex process that requires more than one signal. In addition to TCR binding to peptide-loaded MHC, several co-stimulatory signals are required for full T-cell activation. An appropriate amount of co-stimulation from either B7-1 (CD80) or B7-2 (CD86), expressed on APCs and binding to CD28 on T cells, is required for activating naive T-cells which then leads to IL-2 production. Stimulatory signals from CD28:B7 also lead to the localisation of CTLA-4 to the surface of T-cells (Masteller et al., 2000). Even though CTLA-4 is a homologue to CD28, it has a higher affinity towards B7 providing competitive binding that results in decreased CD28:B7 interactions (Figure 5). The interaction between CTLA4 and B7 does not produce a stimulatory signal required for naive T-cell activation (Parry et al., 2005). Some data has suggested that CTLA-4 has signalling capabilities able to counteract CD28:B7 stimulatory signals. Other inhibitory mechanisms have also been proposed such as direct inhibition at the TCR immune synapse (Parry et al., 2005) or causing an increased T-cell mobility causing a decreased contact frequency with APCs (Schneider et al., 2006). Thus, CTLA-4 is seen as an inhibitor of the co-stimulation usually supplied by the interaction of CD28 and B7. Whether a naive T-cell undergoes activation or anergy is dependent on the balance between CD28:B7 and CD28:CTLA4 signalling. What determines this balance still remains a mystery but multiple mechanisms such as ligand competition between B7 and CTLA-4, regulatory cytokines and CTLA-4 signalling has been proposed (Sansom, 2000). When the balance is tilted towards the negative CD28:CTLA-4 signalling, IL-2 production is halted preventing cell cycle progression (Krummel and Allison, 1996).

As previously mentioned, CTLA-4 is upregulated on the surface of naive T-cells after CD28:B7 or TCR:MHC binding. Before such stimulation is provided, CTLA-4 is present in the cytoplasm of the cell within vesicles (Linsley et al., 1996). CD28 and TCR stimulation causes the exocytosis of the CTLA-4-containing vesicles, leading to the upregulation of CTLA-4 on the surface. This process is under a positive and graded feedback loop where stronger TCR and CD28 stimulation increases CTLA-4 translocation.

The importance of CTLA-4 in maintaining homeostasis was shown in adult mice, where abrogating CTLA-4 expression caused a systemic inflammation and formation of organ-specific autoantibodies. Moreover, congenital CTLA-4 deficient mice died due to lymphoproliferation (Walunas et al., 1998). Similar observations were also shown in humans where patients with CTLA-4 deficiencies suffer from various autoimmune and autoinflammatory diseases. CTLA-4 is not only expressed on naive T-cells but also on regulatory T cells (Tregs). Unlike in naive T-cells, CTLA-4 is constitutively expressed on Treg cells (Takahashi et al., 2000). This constitutive expression of CTLA-4 makes Treg cells key players in maintaining peripheral tolerance. For example, mice with Treg cells with impaired CTLA-4 had impaired suppressive functions (Walunas et al., 1998).

##### 2.2.1.2 CTLA-4 inhibiton for the treatment of cancer

The rationale behind inhibiting CTLA-4 for treating cancer is not a novel idea but has been reported back in 1996 (Leach et al., 1996). Using preclinical models, it was shown that the blockade of CTLA-4 led to anti-tumour immunity. Mice administered with CTLA-4 antibodies rejected pre-established or injected tumours. Moreover, the rejection resulted in immunity against a second tumour challenge. This was further supported by other studies where administering CTLA-4 antibodies to mice with a pre-established B16-BL6 melanoma resulted in tumour clearance (van Elsas et al., 1999). Based on such preclinical evidence, two CTLA-4 antibodies, ipilimumab and tremelimumab, were developed and entered clinical development. Despite acceptable tolerance and durable responses in patients (Ribas et al., 2005; Hodi et al., 2008; Ribas et al., 2013), tremelimumab did not show statistical significance in overall survival (OS) in a phase III trial with advanced melanoma patients128. However, it is disputed that this may have been due to the crossing over of patients from the chemotherapy-only treatment arm to the chemotherapy and tremelimumab treatment arm. Ipililumab on the other hand has been successful in two recent phase III trials with advanced melanoma patients (Hodi et al., 2010; Robert et al., 2011). While the median survival improved minimally, the success of ipililumab was in the remarkable increase in landmark survival after treatment. After 2 years, 18% patients treated with ipililumab in combination with vaccination against the cancer-specific protein gp100 were alive compared to 5% of patients receiving gp100 vaccination alone. In addition, pooled data from clinical trials testing ipililumab in advanced melanoma patients showed that 20% of patients had a long-term survival of at least 3 years (Postow et al., 2015a). Not only confined to advanced melanoma, ipililumab has also succeeded with other malignancies. Pancreatic cancer patients receiving ipililumab had an increase in OS compared to patients receiving chemotherapy only (Le et al., 2013). In addition, it also resulted in responses with prostate cancer patients (Slovin et al., 2013).

While the anti-tumour mechanisms of CTLA-4 antibodies are not well understood, the generally believed hypothesis is that blocking CTLA-4 causes an increased activation of proliferation of effector T-cells accompanied with a decrease in activated Treg cells (Fife and Bluestone, 2008). Supporting this hypothesis, good responses in melanoma patients was attributed to a wide and diverse pool of T-cells (Robert et al., 2014). However, other studies observed that a baseline T-cell diversity, before treatment, was associated with higher OS in metastatic melanoma patients (Khunger et al., 2019). Therefore, pre-existing conditions might be prognostic markers for CTLA-4 blockade anti-tumour efficacy rather than post-treatment induced artifacts*.*

##### 2.2.1.3 PD1/PD-L1 axis

**FIGURE 6:**
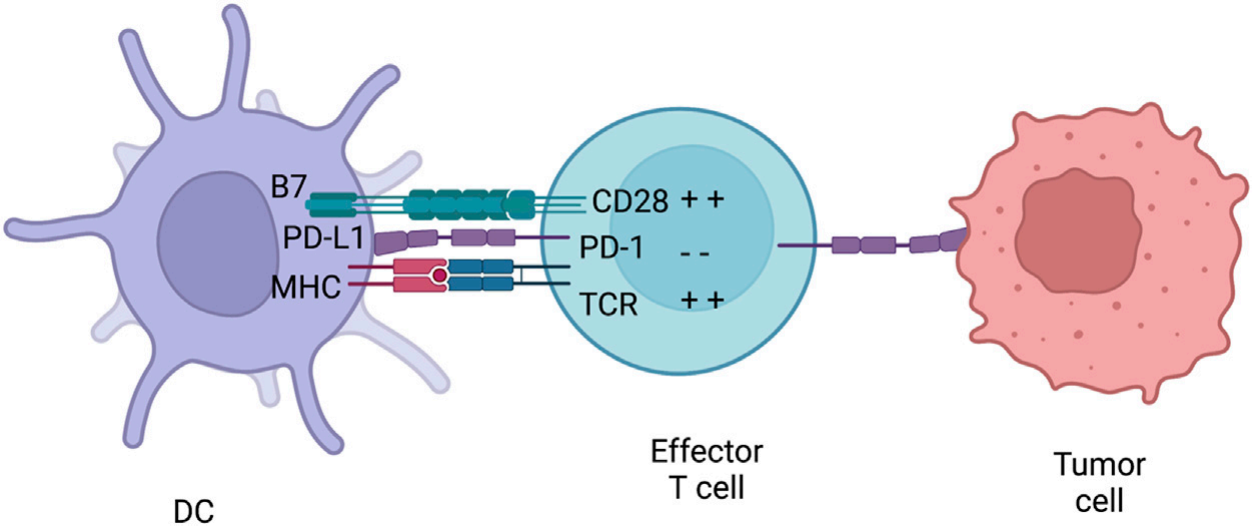
PD-1 inhibition. PD-1 is usually expressed on effector T-cells and binds to either PD-L1 or PD-L2. PD-L1 can be expressed both on immune cells and tumours. Therefore, PD-1 can inhibiteffector T-cells at different stages of an immune response. After PD-L1 is activated by the receptor,they can initiate a signalling complex able to counteract MHC and B7 signalling. Figures were created with BioRender.com.

Similar to CTLA-4, PD-1 is part of the CD28/B7 family of co-stimulatory receptors. It is expressed on effector T cells and regulates them by binding to its ligands, PD-L1 and PD-L2, which are expressed by both hematopoeitic and non-hematopoeitic cells (Figure 6). Activation of PD-1 leads to the phosphorylation of both its intracellular immunoreceptor tyrosine-based switch motif (ITSM) and immunoreceptor tyrosine-based inhibitory motif (ITIM). The phosphorylation of these motifs attracts phosphatases, such as SHP-2, that are able to terminate signalling cascades of both CD28 and TCR (Bardhan et al., 2016). This then inhibits the proliferation and survival of T-cells and production of IL-2, IFN-γ and tumour necrosis factor-α (TNF-α). Hence, PD-1 is able to terminate TCR signalling and reduce T-cell activation. PD-1 is not constitutively expressed on T-cells but rather is a marker of “exhaustion”. After high levels of stimulations from CD4^+^ T-cells, effector T-cells start to express PD-1 in order to prevent over-activation (Wherry, 2011). This exhaustion state is commonly observed both in chronic infections and cancer. Therefore, this causes the suboptimal control of infections and cancer progression. For example, mice chronically infected with cytomegalovirus had virus specific CD8^+^ T-cells present. Yet the T-cells were ineffective since they did not produce cytokines upon antigen challenge. This was also shown in metastatic melanoma patients where exhausted CD8^+^ T-cells were ineffective in tumour clearance.

Expression and the location between PD-L1 and PD-L2 are different. PD-L1 is expressed on many types of tumours and associated with poor prognosis and high TILs (Hino et al., 2010). PD-L2 resides on DC and monocytes but also on non-immune cells depending on the microenvironment. This contrasting distribution of PD-L1 and PD-L2 causes distinct biological effects when each ligand is bound to PD-1. For example, natural killer T cell (NKT) activation under PD-L1 or PD-L2 signalling were opposing (Akbari et al., 2010). Moreover, PD-L1 and CD80 interaction decreased T-cell response unlike when PD-L2 was blocked, an increased T-helper 2 cell (Th2) activity was noted (Huber et al., 2010). These opposing biological effects provide an explanation on the toxicity levels caused by inhibiting PD-1 and has highlighted the use of PD-L1 inhibitors.

Even though CTLA-4 and PD-1 have similar negative effects on T-cells, there are key differences between the checkpoints. CTLA-4 controls the activation of naive T-cells in lymph nodes whereas PD-1 controls T-cells in the effector phase in the periphery tissues. CTLA-4 expression is confined to T-cells, unlike PD-1 that is expressed on T-cells, B-cells and myeloid cells. Furthermore, the expression and distribution of checkpoint ligands differs. B7 is restricted to professional APCs while PD-L1 and PD-L2 are expressed on leukocytes, non-hematopoeitic cells, and non-lymphoid tissues (Keir et al., 2008). These differences indicate that CTLA-4 down regulates T-cell responses early on in an immune response while PD-1 limits T-cell response later during the effector stage. This then causes different effects *in vivo* when each receptor is inhibited*.* Blocking CTLA-4 causes an increase in activation and proliferation of effector T-cells regardless of TCR specificity while PD-1 inhibition leads to restoring proper T-cell functions.

##### 2.2.1.4 Targeting the PD-1 axis in cancer therapy

After the success in targeting CTLA-4 during cancer, many antibodies have been designed to disturb the PD-1 axis for a similar purpose. Although the antibodies differ in structure (antibody isotype and chimerised/humanised), they can be categorised in two main groups: antibodies targeting the PD-1 receptor and antibodies targeting the ligand PD-L1. PD-1 antibodies such as nivolumab and pembrolizumab were shown in a phase I clinical trial with advanced melanoma, non-small cell lung cancer, renal cell carcinoma and other tumours to be tolerable and result in high durable responses (Topalian et al., 2012; Topalian et al., 2014). Recently, results from three phase III clinical trials with advanced melanoma have been published. In all three trials the OS was significantly higher in patients receiving nivolumab. In addition to melanoma, renal cell carcinoma patients treated with nivolumab had an OS of 25 months compared to 19.6 months in patients receiving the current standard treatment, everolimus (mTOR inhibitor). Similar results were also shown in a phase III trial with non-small cell lung cancer. The mechanism behind the anti-tumour effects of PD-1 blockade, which occurs during the effector stage of T-cells, involves re-activating peripheral T-cells that have been “exhausted” due to the high exposure of tumour antigens. Many studies have reported that PD-1 is expressed on TILs (Topalian et al., 2014; Cao et al., 2017). Thus, PD-1 inhibition allows the suppressed TILs to gain back their anti-tumour properties. However, a recent study indicated that ipililumab response was associated with levels of expression of PD-L1 on tumour cells (Taube et al., 2014). When PD-1 is inhibited, the interaction between PD-1:PD-L1 is blocked yet PD-L1 is still able to inhibit T-cells by binding to CD80, a second receptor for this ligand. To overcome such limitation, PD-L1 antibodies have been generated and are able to disrupt both PD-1:PD-L1 and PD-L1:CD80 interactions. These antibodies are also able to keep intact the interaction between PD-1 and PD-L2, required for self-tolerance and thus leading to lower toxicities. Three PD-L1 antibodies (Atezolizumab, Durvalumab and Avelumab) have been clinically approved and have shown durable responses and less toxicity levels in a variety of tumours (Postow et al., 2015b).

##### 2.2.1.5 Fc silencing of checkpoint inhibitors

The Fc-region of antibodies provides the ability to elicit Fc-effector mechanisms which are crucial for clinical efficacy. With respect to checkpoint inhibitors, it has been a subject of debate. For CTLA-4 checkpoint inhibitor, ipilimumab, Fc-effector mechanisms pertinent to the IgG1 isotype has been correlated with clinical efficiency. Advanced melanoma patients with Fc-γIIIA polymorphism V158F increasing IgG1 affinity, have been shown to respond better to ipilimumab (Vargas et al., 2018). Silencing Fc-effector mechanisms by changing the Fc-isotype or adding point mutations have seen to reduce *in vivo* activity of CTLA-4 inhibitors. The mechanism behind this has been argued to be the depletion of immunosuppressive Treg populations. However, opposite results were seen with PD-1 checkpoint inhibitors where Fc-effector mechanisms have lowered the *in vivo* anti-tumour activity (Dahan et al., 2015). PD-1 checkpoint inhibitors with competent Fc regions able to elicit Fc-effector mechanism were shown to deplete crucial CD8+T cell and CD4^+^ T cell populations (Dahan et al., 2015). These results explain why all of the PD-1 checkpoint inhibitors approved in the clinic are of the IgG4 isotype, an isotype that elicits low levels of ADCC and CDC, and have a S228P mutation decreasing Fc-γR binding. This reduction in Fc-effector activity then prolongs CD8+T cell binding which subsequently increases anti-tumour activity.

As for PD-L1 checkpoint inhibitors, a safety concern over the addition of Fc-effector mechanisms exists. This is because PD-L1 expression is not solely limited to tumor cells but also can be expressed on healthy cells149. In result, the opsonisation of healthy cells with an antibody able to elicit Fc-effector mechanism can be deleterious. Out of the three approved PD-L1 inhibitors, atezolizumab and durvalumab have point mutations in the IgG1 Fc-region that remove Fc-γR binding. However, *in vivo* data has shown that arming such checkpoint inhibitors can increase anti-tumor efficacy (Dahan et al., 2015; Yi et al., 2021). This was attributed to an increased clearance of tumor cells but also immunosuppressive immune populations. Therefore, a strategy to increase Fc-effector mechanism while maintaining safety concerns is required.

#### 2.2.2 Cancer vaccines

Vaccines have been a major milestone in preventing life threatening infectious diseases. The concept of being able to induce an immune response resulting in a protective immunological memory against cancer is ideal. Not only could this prevent or treat cancer but also help in tumour relapse. Nevertheless, cancer genomics has shown the complexity in achieving this since most of the tumour antigens being highly expressed on tumours are also shared among healthy cells. TAAs such as HER-2, glycoprotein (gp) 100, Telomerase and others are ideal antigen candidates due to their immunogenic properties, yet are expressed on healthy tissue (Finn, 2018; Blass and Ott, 2021). This lack of specificity is concerning due to the “off-target” effects that can be very toxic to a patient. However, in 2010 the first therapeutic cancer vaccine, Sipuleucel-T, was approved by the FDA for asymptomatic or mildly symptomatic metastatic prostate cancer (Plosker, 2011). This vaccine consisted of isolating dendritic cells from patient’s PBMCs and expanding/activating them *ex-vivo* using the commonly known TAA called prostatic acid phosphatase (PAP). The approval of such cancer vaccine stimulated other vaccine platforms to be investigated in the clinic. For example, BioNTech have developed a novel RNA lipoplex complex, called FixVac, coding for different TAAs (Sahin et al., 2020). Such platforms can selectively target dendritic cells to induce an appropriate antigen presentation allowing for an effective T-cell immune response.

The perfect type of cancer vaccine would include an antigen selectively expressed on tumour cells. The genome of cancer cells is unstable and undergoes many genetic modifications such as somatic mutations, deletions, duplications and other processes. Due to such instability, neoantigens arise from cancer cells that are not found in healthy cells. Hence, such antigens then represent ideal targets for cancer vaccines. Nonetheless, these antigens are not very immunogenic and fail to induce a sustainable immune response. The first clinical trial evaluating a neoepitope based vaccine was with stage III cutaneous melanoma patients. These patients were injected with A*02:01-specific neoepitopes and a specific CD8^+^ T cell response was observed (Lee et al., 2021). Yet, this activation was modest and was not very effective in controlling tumor growth. This field is still a hot topic with multiple type of strategies trying to further strengthen immunogenicity.

#### 2.2.3 Oncolytic viruses

Scientists have stopped hunting for individual tumor suppressor genes or oncogenes and started investigating methods in disrupting whole tumorigenic biological pathways. Oncolytic viruses (OV) are the ideal agents in achieving this. Such viruses are able to thrive in tumor cells where such malignant pathways have been activated or disrupted, and exploit metabolic pathways that characterize tumorigenesis which result in oncolysis (Lawler et al., 2017). Also, oncolytic viruses have extensively been shown to stimulate systemic host immune responses (Lemos de Matos et al., 2020). The tumor microenvironment is immunosuppressive and boosting the immune system has been observed to have significant anti-tumor effects (Zhao et al., 2021). Hence the dual mechanism OV possess makes them interesting therapy agents.

These viruses have been genetically modified to conditionally replicate in cells in which specific cellular pathways are disrupted. This then allows OVs to infect both healthy and tumor cells and only replicate in tumor cells in which cellular pathways are compromised, but be recognized and cleared by healthy cells by the intrinsic immune system. However, studies using immunocompetent and immunocompromised mice have shown that the direct oncolysis of such viruses is not enough to induce tumor clearance (Grote et al., 2001). After tumor lysis, various TAAs are released and made accessible by nearby DCs (Hollingsworth and Jansen, 2019). Such TAAs are then able to be taken up, processed and presented on MHC complexes allowing for adaptive tumor-specific tumor responses to be formed. This mechanism of action has been shown to be key for a successful response. Many different types of DNA and RNA oncolytic viruses have and are currently under clinical development and testing. In this review, a specific focus will be drawn into adenoviruses to be used as OVs.

## 3 Adenoviruses and their roles as cancer therapies

### 3.1 Adenoviruses

Adenoviruses were first discovered when scientists were investigating adenoid cells (Rekosh et al., 1977). They observed that these viruses featured a double-stranded DNA genome of about 36 kb packaged into a capsid with an icosahedral shape. Adenoviruses are medium sized, around 100nm, particles with a non-envelope capsid composed of a penton, hexon and fiber knob domain all required for attachment and entry. There has been 57 different serotypes identified to date which can be subdivided into 6 groups (A-F).

Unlike many other viruses, adenoviruses circulate through humans during the whole year and are endemic in children. The mode of transmission of the virus is through water and fomites. Owing to their success of infection, adenoviruses are resilient to harsh environments due to their resistance to chemical and physical agents. For example, the resistance to gastric acid and biliary secretions has allowed such viruses to infect the gastrointestinal tracts (Hierholzer, 1992). Moreover, adenoviruses can withstand being outside the host for up to 3 weeks. To our advantage, despite causing flu-like symptoms these viruses rarely induce serious disease in healthy human but can be generate illness in immune-compromised patients. No animal reservoirs have been identified making the virus hard to study due to low animal models to mimic disease (Ginsberg and Prince, 1994).

The infection process starts off with the protein-interactions between the adenovirus capsid and host-cellular membranes. The adenovirus capsid is comprised of 240 hexons and 12 pentons. Other minor components such as pIX, pVIII, pVI and IIIa are also present in the capsid. The pentons, which consists of complex of five polypeptide III, provide the base for the trimeric fiber to attach. The fiber contains the knob-fiber domain that is then responsible for attaching to host-cellular membranes. The receptor that the fiber binds to depends on the serotype but the main ones include the coxsackie adenovirus receptor, desmoglein-2 or CD46 (Bergelson et al., 1997; Wu et al., 2004; Marttila et al., 2005; Wang et al., 2011). After initial binding, the pentons in the adenovirus interact with the host-cell integrins (α_v_β_3_ or α_v_β_5_), leading to activation of certain signaling proteins (GTPases, phosphoinositide-3-OH kinase and MAPK) which induce the uptake of the virus particles *via* clathrin-coated vesicles (Wickham et al., 1993).

Once inside the vesicles, the acidification of the endosome cues for dismemberment of the viral capsid by proteolytically cleaving protein VI(171). After endosomal escape, the resulting virion is released and transported to the nucleus with the help of dynein and microtubules which interact with capsid proteins (mu, proteins VII and V). Once the adenovirus genome reaches the nucleus, the transcription of genes begins and is divided into two phases; early and late. The early phase consists in the transcription and translation of early gene products (E1A, E1B, E2, E3 and E4) which help in the replication of the adenovirus DNA genome. Moreover, the products of the early genes also then induce the expression of late genes (L1, L2, L3, L4 and L5). The late gene products are required for virion assembly since they represent the structural proteins.

After the genome has been replicated and the structural proteins expressed, virion assembly begins with the hexons and pentons clustering with multiple scaffolding proteins (L4 22-33K) (Ahi and Mittal, 2016a). This induces the insert of the viral DNA inside the virion structure and the final maturation of the virus by the release and cleavage of precursor proteins (L1 52-55K) (Ahi and Mittal, 2016a). The whole replication process of the adenovirus usually takes 24–36 h and can yield about 10,000 virions per cell to be released (Giberson et al., 2012).

#### 3.1.1 Adenovirus genome, replication, and machinery behind it

Despite the small genome of 30–36 kb in length, adenoviruses can encode for multiple genes due to the overlapping open reading frames, alternate splicing, and ability of transcription from both strands of the genome. (Hoeben and Uil, 2013). As described previously, the early gene products are responsible for genome replication and mainly consist of the preterminal protein (pTP), DNA polymerase (Ad Pol) and DNA-binding protein (DBP). The late genes of the adenovirus include proteins involved in virion assembly and encapsulation and are only expressed once the early genes are. The multiple late genes are usually arranged in the adenovirus major late transcription unit (MLTU) which consists of five regions, L1-L5, and are under transcriptional control of the major late promoter (MLP) (Berget et al., 1977; Chow et al., 1977; Nevins and Darnell, 1978; Nevins and Chen-Kiang, 1981; Ramke et al., 2017). Other than the early and late genes, the adenovirus has also two other gene products, pIX and IVa2, which are often described as intermediate genes since they are not in the MLTU but facilitate the expression of the late genes.

The adenovirus genome is flanked by inverted terminal repeats (ITR) which compromise of around 100 bp each. These ITRs contain a ∼50 bp origin of replication which is made up of a core origin and auxiliary origin (Charman et al., 2019). The core region provides the binding site to pTP and and Ad Pol while the auxiliary region provides for cellular transcription factors nuclear factor 1 (NF1) and OCT-1 (De Jong et al., 2003). Moreover, near the ITR regions the adenovirus genome has a packaging sequence (ψ) which is required for encapsulation in virions (Ostapchuk and Hearing, 2003; Ahi and Mittal, 2016b). Finally, to the 5’ ends of the genome terminal proteins (TP) is covalently attached which protects the DNA from degradation.

**FIGURE 7:**
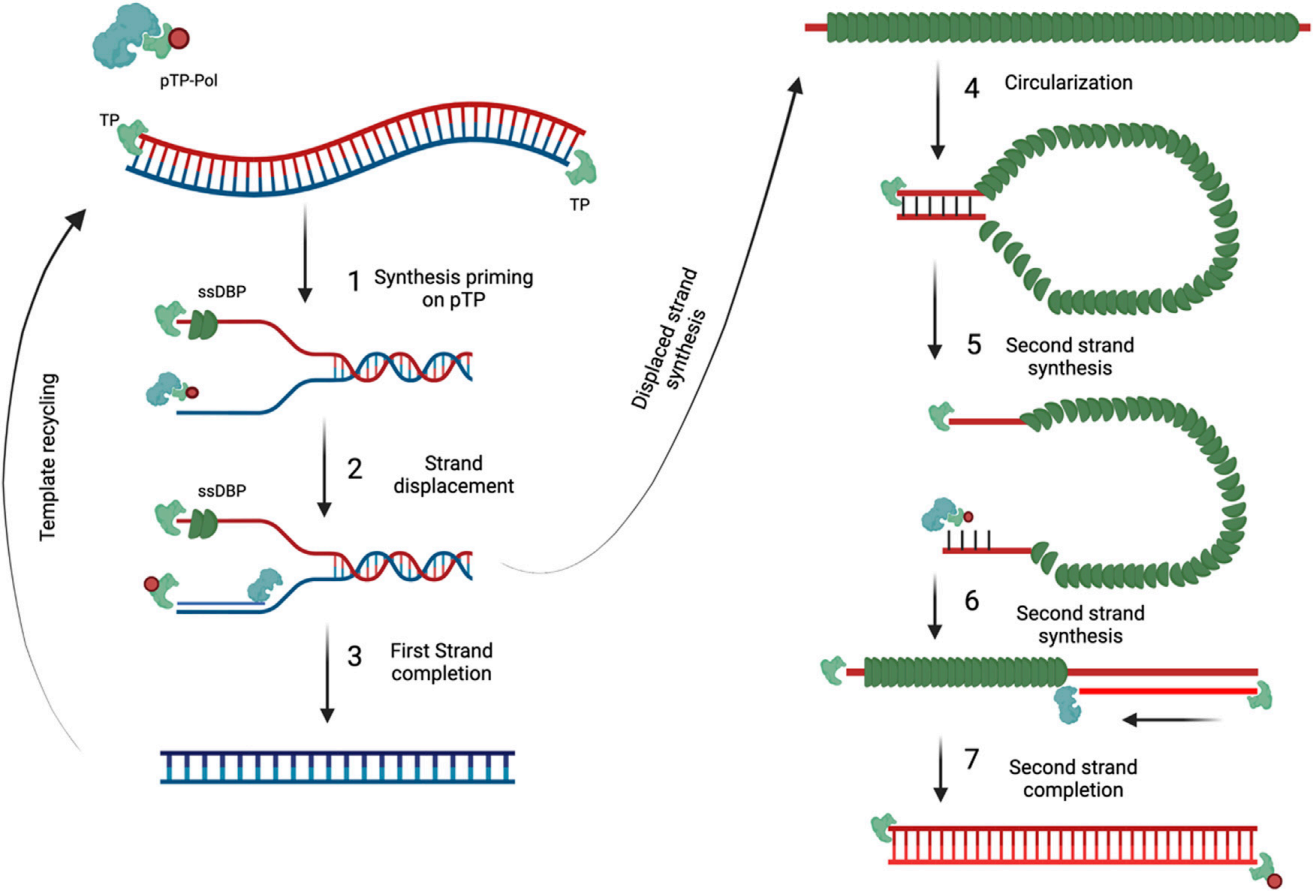
Adenovirus genome replication. DNA replication begins with the pTP-Pol complex invadesand serves as a primer to begin DNA replication (1). Pol protein then begins to synthesize the newDNA strand and displaces the original strand (2) which is then coated with DBP. As soon as the firststrand is completed it can then be used for template recycling (3). The displaced strand covered inDBP then circularizes because of the complementary ITR regions (4). The circularized DNA strand isthen used as a template and evaded by the pTP-Pol complex to begin the synthesis of thecomplementary strand (5 and 6) until completion (7). Figures were created with BioRender.com.

Genome replication starts with the formation of the pre-imitation complex which consists of multiple protein-protein and protein-DNA interactions (Figure 7). Firstly, Ad Pol will covalently attach to pTP *via* the dCMP nucleotide on its S580 amino acid position (Desiderio and Kelly, 1981; Smart and Stillman, 1982). Following Ad pol binding, DBP then binds to the core origin which then further facilitates the binding of Ad Pol and NF1 to core origin and auxiliary origin, respectively. NF1 and OCT-1 are not necessarily required for genome replication but rather enhance replication. After, Ad Pol dissociates from pTP and the formation of the nascent strand can then begin. This is marked with the dissociation of the pre-initiation complex and allowing DBP to unwind the dsDNA and allowing Ad Pol to form the nascent strand. Interestingly, displaced ssDNA can anneal to itself *via* the intramolecular/intermolecular interactions of the ITR regions which create dsDNA origins of replications. Hence, both dsDNA and ssDNA can be used as replication intermediates to increase the genome copy number.

#### 3.1.2 Adenovirus-host cell interactions and selective replication

There exist very important interactions between viral and cellular-host proteins that facilitate the adenoviral replication cycle. During the replication cycle of adenovirus, the virus must sequester various cellular proteins to help in genome replication, transcription, and translation. Moreover, while doing so it also has to fight off the intrinsic pathways of the host cell that are designed to shut off cell machinery, induce apoptosis and clear the virus. Therefore, adenoviruses have multiple proteins aiding in facilitating all these processes. Due to the understanding of such mechanisms, scientists have been able to come up with genetic modification allowing adenovirus to conditionally replicate in tumor cells (Heise and Kirn, 2000). This section will describe these crucial interactions and how scientists have taken advantage of them to create conditionally replicating adenoviruses (CrAd).

For the adenovirus to start replicating its genome, the cell must be directed into S-phase (Sha et al., 2010). The adenovirus expresses E1a protein which is responsible in doing so by interacting with retinoblastoma protein (pRb). Under normal conditions, pRb can control the cell-cycle by interacting with DNA-binding transcription factor E2F (Heise et al., 2000). This interaction restricts E2F from binding to DNA and promoting cell replication. E1a can bind to pRB and restrict its interaction with E2F(Hemminki et al., 2015). This then leads to E2F to be dissolved and bind freely to DNA and promoting transition into S-phase. Usually in malignant cells, the mechanisms controlling cell replication are defective to sustain cell growth. The majority of cancers have a deficient pRb protein and consequently an E2F roaming freely (Wu and Wu, 2021). In consequence, adenoviruses do not require E1a to replicate in tumor cells and becomes non-essential. Therefore, removing or rendering E1A defective can lead to a selective replication of adenoviruses in tumor cells (deficient in pRb proteins) while unable to replicate in healthy cells due to pRb. A 24 base pair deletion in the E1A protein has been previously described causing the protein unable to bind to pRb (Hemminki et al., 2015). Various clinically tested oncolytic adenoviruses apport this mutation making them selectively replicate in tumor cells and have been shown to be safe.

After adenovirus infection pushes cells into the S-phase, p53 accumulates as a response to induce apoptosis to control cell growth (Lomonosova et al., 2005). To circumvent this, adenoviruses express E1B 55k, E4 orf6 and E1B 19K which are all able to interact with p53 directly or indirectly to avoid apoptosis (Piya et al., 2011). Nevertheless, apoptosis might be disrupted but E1B 19K induces autophagy at the end of the viral replication cycle to release virions. Like pRb, p53 is also mutated in most cancers which prompts for a different strategy to induce selectivity in replication for adenoviruses. Deletion of the E1B gene induces adenoviruses to replicate in tumor cells while leaving healthy cells free (Cheng et al., 2015). This deletion has also been noted to be clinically safe with different oncolytic viruses, such as ONYX-015 and H101, and in some countries they have been approved as therapy (Heise et al., 1997; Cheng et al., 2015).

#### 3.1.3 Arming oncolytic adenoviruses

**FIGURE 8:**
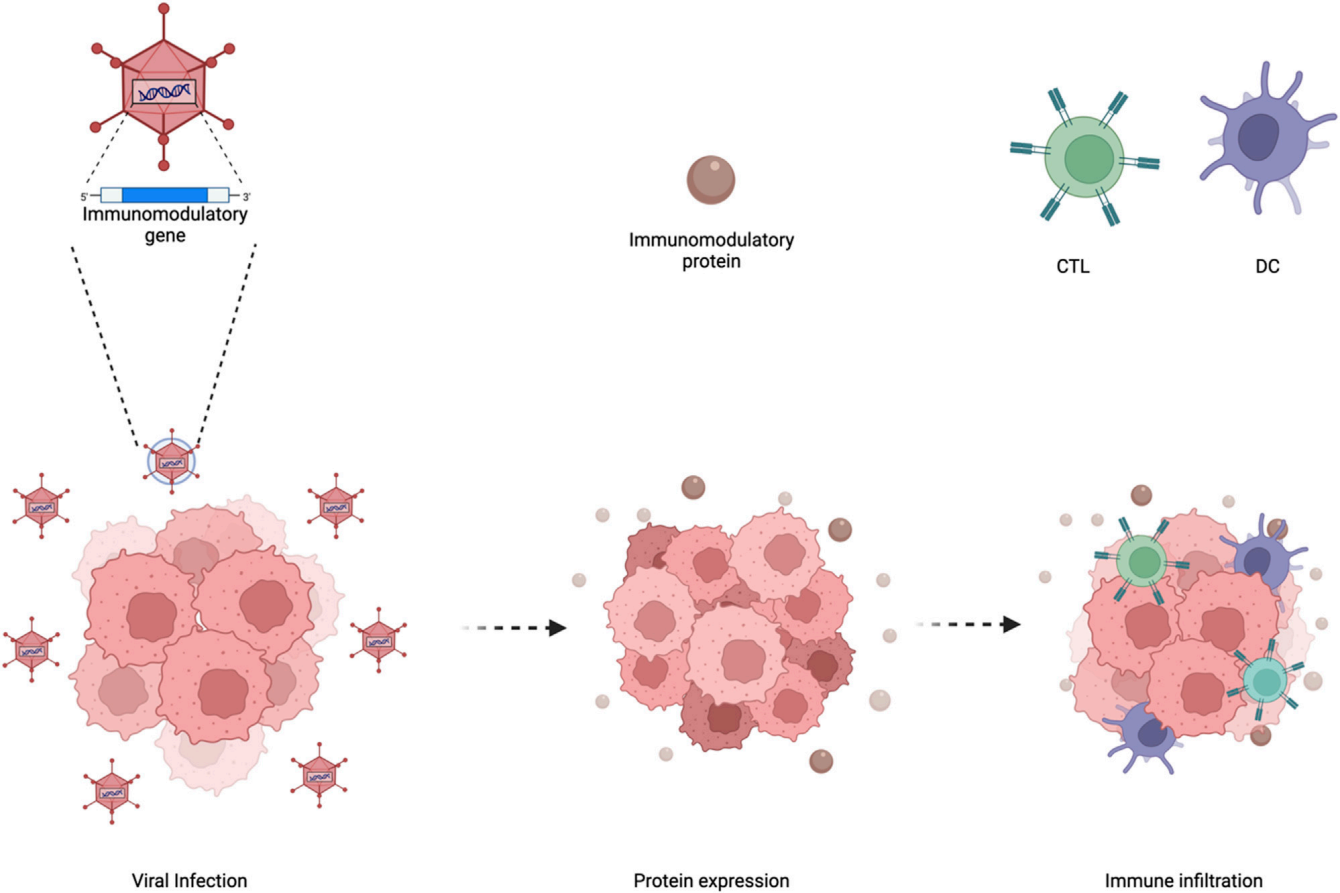
Enhancing oncolytic adenoviral therapy. Oncolytic adenoviruses have a specific tumortropism which can be utilized to arm such viruses with immunomodulatory genes. This then leads tothe expression and secretion of the immunomodulatory proteins in the tumor microenvironmentwhich increases immune infiltration. Figures were created with BioRender.com.

As mentioned previously, even though oncolytic adenoviruses can directly infect selectively tumor cells and induce oncolysis this is not enough for clinical efficacy. The release of TAAs from oncolysis leading to a vaccination effect is required for a successful treatment. However, a major limitation from achieving such clinical efficacy is the absence of anti-tumor immune cells and/or preventing their anti-tumor functions. One of the key advantages of using oncolytic adenoviruses is the ability to turn “cold” tumors with poor immune infiltration into “hot” tumors with high immune infiltration (Bramante et al., 2015). Yet, the amount of immune stimulation provided seems not to be enough to sustain clinical efficacy or tumor elimination. Despite this, researchers have armed oncolytic adenoviruses with various molecules ranging from cytokines, antibodies, bi-specific antibodies (BiTEs) and more (Figure 8). Other than expressing adequate levels of immunomodulatory molecules, the oncolytic tropism of the virus may help in circumventing toxicity issues by limiting expression in the tumor microenvironment with minimal leakage to the periphery.

One class of molecules that has been used to arm oncolytic adenoviruses are co-stimulatory molecules. An example is the arming of oncolytic adenoviruses with two immune-activating ligands CD40L and OX40L (Malmström et al., 2010; Pesonen et al., 2012; Loskog et al., 2016; Schiza et al., 2017). CD40L when secreted can interact with CD40 present on APCs and enhance their antigen presentation and co-stimulation capacity (Piechutta and Berghoff, 2019). Moreover, OX40L binds to OX40 found on T cells and induces the survival and homeostasis of memory T-cells (Croft et al., 2009). Another strategy was arming oncolytic adenovirus LOAd703 with CD40L and 4-1BBL (Eriksson et al., 2017). The interaction of 4-1BBL with 4-BBL among T cells and APC lead to the increase of T-cell proliferation and activation. LOAd703 has been tested in clinical trials against many solid tumors and, interestingly, with pancreatic cancer it has been seen to reduce myeloid derived suppressed cells (MDSC) and increase memory T cells in many patients.

The release of cytokines and chemoattractant from oncolytic viruses are a successful strategy to increase immune cell homing to the tumor. The only FDA approved oncolytic virus in the clinic, T-VEC, compromises of a herpes simplex virus expressing GM-CSF (Liu et al., 2003). This cytokine helps in the maturation and antigen presentation of APC, leading to better induction of T-cell immune responses. A similar version of T-VEC exists, but rather than a herpes simplex virus an adenovirus is used with the 24 base pair deletion in its E1A, previously described, to express GM-CSF (Cerullo et al., 2010). Such virus, called ONCOS-102, is under clinical evaluation and was seen to increase CD8+T cells circulation but more importantly antigen-specific CD8^+^ T cells in mesothelioma and multiple peritoneal malignancies (Ranki et al., 2016). Many oncolytic adenoviruses have been used to locally express various cytokine such as IL-2 and tumor necrosis factor α (TNF-α), IL-18, IL-24 or IL-12 in order to potentiate anti-tumor immune responses. Other than cytokines, chemokines such as CXCL9 and CXCL10 have also been cloned in oncolytic adenoviruses to recruit T-cells (Ylösmäki and Cerullo, 2020).

BiTEs are small molecules which consist of two scFv directed at different tumor antigens. Convential BiTEs usually have one of their scFV directed towards CD3 while the other towards a TAA. These BiTE’s main mechanism of action is bringing CD3^+^ T cells into close proximity of tumor cells and induce MHC-independent killing (Scott et al., 2018). These BiTEs have been shown to be excellent therapies for the treatment of lymphomas and leukaemias. For example, Blinatumomab, against CD3 and CD19, is the first BiTE to be approved by the FDA for the use of B-maligancies (Goebeler and Bargou, 2016). However, for solid tumors it has been seen not to be effective since their half-life in blood is short-lived, which consequently requires constant infusion of treatment leading to systemic toxicities. Yet, oncolytic adenoviruses have shown to provide excellent platforms to deliver BiTEs locally and persistently in solid tumors. Enadenotucirev is one of the first oncolytic adenoviruses to express a BiTE, which was directed towards TAA epithelial cell adhesion molecule (EpCAM) and CD3 (Yu et al., 2014). Other than just targeting TAA, a similar oncolytic adenovirus expressing BiTE was also constructed but directed towards fibroblast activation protein (FAP) which is found on cancer-associated fibroblasts (Freedman et al., 2017). The combination of both viruses demonstrated enhanced anti-tumor efficacy and T cell recruitment and function.

The systemic administration of checkpoint inhibitor has been associated with many adverse events. To further improve the safety profile, checkpoint inhibitors have been packaged into the genome of oncolytic adenovirus. Checkpoint inhibitors against CTLA-4 (Dias et al., 2011) and PD-L1 (Wang et al., 2020) have been cloned into oncolytic adenoviruses and have shown to be effective in controlling tumor growth with a high safety profile. Yet, a limiting factor that needs to be addressed with this strategy is that adenovirus has low capacity for cloning long transgenes in the genome. Hence, cloning whole antibodies consisting of a heavy and light chain can affect the viral fitness.

#### 3.1.4 Construction of adenoviral vectors

The use of adenoviruses for gene therapy, vaccines and cancer immunotherapies has increased throughout the years. This entails the engineering of adenovirus to express any gene of interest (GOI), a process that has been modified several times in order to optimize the procedure. The classical approach that many scientists have used, is the cloning the GOI in a shuttle plasmid containing a 5′-ITR, a packaging signal and sequence of homologous recombination (Stratford-Perricaudet et al., 1992; Mittal et al., 1993). This shuttle plasmid is then transfected into HEK293 cells with an adenovirus vector for homologous recombination to occur and create an adenovirus genome incorporating the GOI. Another used method is the cloning of the GOI into a similar shuttle plasmid but the homologues recombination sequence is substituted with LoxP site(s) (Hardy et al., 1997). This shuttle is then transfected into HEK293 cells with an adenovirus genome containing LoxP sites. The shuttle vector and adenovirus genome are then joined *via* Cre recombinase-mediated recombination. A separate cloning method is the use of shuttle vector containing a 5′-ITR, a packaging signal and sequence of homologous recombination flanking a kanamycin resistance gene. After the GOI is added to this shuttle, it is linearized and transfected into bacterial cells (BJ5183) along with an ampicillin-resistant adenovirus backbone (He et al., 1998). Colonies are then screened based on kanamycin resistance and the final adenovirus containing the GOI product is linearized and transfected into HEK293 cells.

Each cloning system presented here are well characterized, reproducible and easy to design and carry out. However, these methods are very time consuming, and it can take up to 6 months or more to obtain the final product. This is because homologous recombination has very low efficiency and can take multiple rounds for a positive colony. Moreover, secondary recombination can occur leading to the incorporation of unwanted repeated regions or secondary structures. Hence, the need of novel cloning methods that are faster, easier and reliable are required.

## 4 Conclusions and future prospects

**FIGURE 9:**
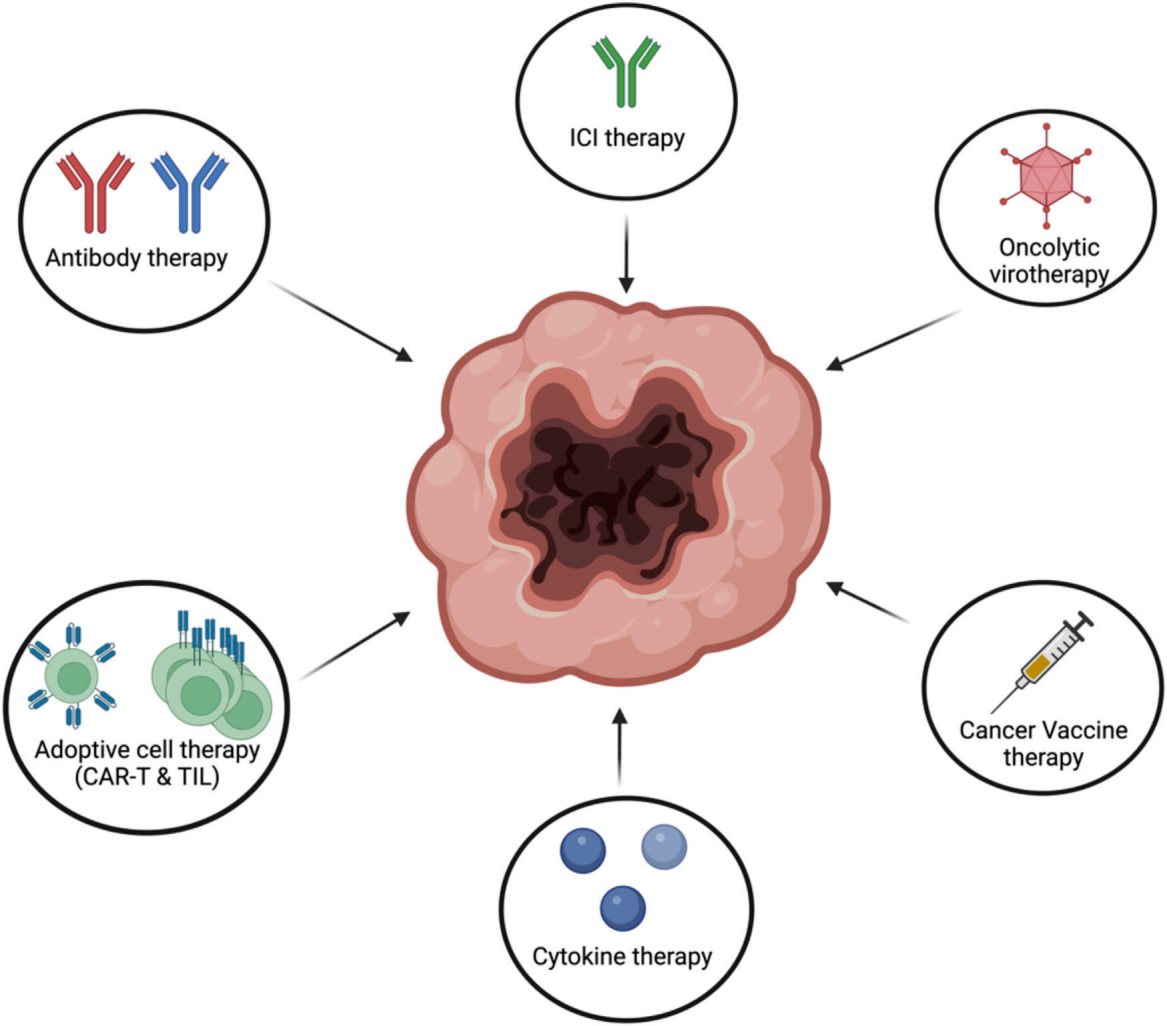
Different types of cancer immunotherapies. Cancer immunotherapies can come in differentfrom such as antibody therapy, ICI, oncolytic virotherapy, cancer vaccines, adoptive cell therapy orcytokine therapy. Figures were created with BioRender.com.

Cancer immunotherapies have taken the main stage in the treatment of cancer. This is mostly due to the dramatical increase in survival and quality of life for cancer patients. Nevertheless, since cancer is heterogenous not one type of cancer immunotherapy works for all. Depending on multiple factors, certain cancer immunotherapies work better for some cancer patients than others (Figure 9).

To date, only one oncolytic virus has been granted FDA approval for treatment despite years of extensive investigation. One of the reasons is that oncolytic viruses have been generally seen as direct tools for killing cancer due to their tumor-specific tropism. A growing body of evidence has shown that the ability of the virus to activate the immune system is a key attribute with regard to long-term antitumor effects. Therefore, to make more significant advances with such therapies there has been a shift in focus from not solely viewing oncolytic viruses as direct oncolytic tools but also as immunotherapies. This further validates the importance of harnessing the immune system to combat cancer rather than using cytotoxic drugs. Cientists have equipped oncolytic viruses with multiple immune-stimulatory molecules which have enhanced anti-tumor effects. Other than enhancing anti-tumor effects, this has also had a positive effect regarding limiting toxicities since the expression/release of the molecules is limited to the tumor.

Cytokine therapy is an effective strategy to induce an anti-tumor immune response to combat cancer. Yet, safety is one of the main limitations that made clinical approval hard to obtain for cytokine therapy. Usually, these molecules are injected systemically and can induce an overwhelming immune activation leading to various immune-related side effects. One strateg that has been used to circumvent this has been the use of biological carriers, like OV, to limit the immune activation in the tumor microenvironment. Moreover, due to the poor infiltration of CAR-T cells to solid tumors the, the combination of cytokine therapy and CAR-T cells has been tested. Research has shown that cytokine therapy can stimulate higher tumor infiltration of CAR-T cells with solid tumors. These observations further emphasize the testing of different combinations of cancer immunotherapies as possible synergism might exist. Another example of a possible synergy is between cancer vaccines and ICI therapy. Cancer vaccines have been shown to orchestrate an effective T-cell immune response specific towards TAAs. The combination with ICI’s, specifically PD-1 or PD-L1 inhibitors, could further enhance the anti-tumor T-cell response by decreasing the inhibitory effects exerted by the tumor cells to escape immune desctruction.

Almost all the cancer immunotherapies in the clinic target PBMCs. Many studies have shown that targeting solely PBMCs does not cause full clearance since the cytotoxic effects mediated are finite. PMNs have been a neglected cell population despite being the largest leukocyte population in blood and highly infiltrated in tumors. This has been mostly due to the use of IgG antibodies which sub-optimally activates neutrophils. This is simply because PMNs highly express CD32b and CD16b which downregulate effector functions or act as a molecular “sink”, respectively. This can be dangerous since immune cells have shown to be malleable depending on the microenvironment and stimulus provided. For example, researchers found relatively normal levels of Treg cells in the synovial membrane from rheumatoid arthritis patients compared to healthy individuals. However rather than promoting immune resolution, Tregs cells from the patients were programmed to secrete a powerful pro-inflammatory cytokine IL-17. The mechanism behind was speculated to be most probably due to the influence of the highly inflammatory microenvironment. Despite this, it could explain why the infiltration of neutrophils to the tumor is associated with a lower prognosis since they are not adequately activated (Masucci et al., 2019). This then calls for appropriate molecules able to capitalize such population to be used as an effector population. Moreover, rather than just activating PMNs the main goal in the future would to also involve other effector mechanisms. Multiple studies have shown that involving more than effector population leads to higher tumor killing compared to when each population is used on its own.
